# Regular physical exercise before entering military service may protect young adult men from fatigue fractures

**DOI:** 10.1186/s12891-019-2513-4

**Published:** 2019-03-25

**Authors:** Harri Pihlajamäki, Mickael Parviainen, Heikki Kyröläinen, Hannu Kautiainen, Ilkka Kiviranta

**Affiliations:** 1 Department of Orthopaedics and Traumatology, Seinäjoki Central Hospital, Seinäjoki and University of Helsinki, Helsinki, Finland; 2 Mehiläinen Medical Centre, Helsinki, Finland; 3 Neuromuscular Research Center, Faculty of Sport and Health Sciences, University of Jyväskylä, Jyväskylä, Finland; 4 National Defence University, Helsinki, Finland; 5 Unit of Primary Health Care, Helsinki University Central Hospital, Helsinki, Finland; 6 Department of General Practice, University of Helsinki, Helsinki, Finland; 7 Unit of Primary Health Care, Kuopio University Hospital, Kuopio, Finland; 8 Department of Orthopaedics and Traumatology, University of Helsinki and Helsinki University Hospital, Helsinki, Finland; 9 Mediwest Research Center, Koskenalantie 16, 60220 Seinäjoki, Finland

**Keywords:** Stress fractures, Fatigue fractures, Physical activity, Military training, Epidemiology, Injury prevention, Incidence, Exercise

## Abstract

**Background:**

Bone stress fractures are overuse injuries commonly encountered in sports and military medicine. Some fatigue fractures lead to morbidity and loss of active, physically-demanding training days. We evaluated the incidence, anatomical location, risk factors, and preventive measures for fatigue fractures in young Finnish male conscripts.

**Methods:**

Five cohorts of 1000 men performing military service, classified according to birth year (1969, 1974, 1979, 1984, 1989), were analysed. Each conscript was followed for his full military service period (180 days for conscripts with rank and file duties, 270 days for those with special training, 362 days for officers and highly trained conscripts). Data, including physical activity level, were collected from a standard pre-information questionnaire and from the garrisons’ healthcare centre medical reports. Risk factor analysis included the conscripts’ service class (A, B), length of military service, age, height, weight, body mass index, smoking, education, previous diseases, injuries, and subjective symptoms, as well as self-reports of physical activity before entering the service using a standard military questionnaire.

**Results:**

Fatigue fractures occurred in 44 (1.1%) of 4029 men, with an incidence of 1.27 (95% confidence interval: 0.92–1.70) per 1000 follow-up months, and mostly (33/44, 75%) occurred at the tibial shaft or metatarsals. Three patients experienced two simultaneous stress fractures in different bones. Most fatigue fractures occurred in the first 3 months of military service. Conscripts with fatigue fractures lost a total of 1359 (range 10–77) active military training days due to exemptions from duty. Conscripts reporting regular (> 2 times/week) physical activity before entering the military had significantly fewer (*p* = 0.017) fatigue fractures. Regular physical activity before entering the service was the only strong explanatory, protective factor in the model [IRR = 0.41 (95% CI: 0.20 to 0.85)]. The other measured parameters did not contribute significantly to the incidence of stress fractures.

**Conclusion:**

Regular and recurrent high-intensity physical activity before entering military service seems to be an important preventive measure against developing fatigue fractures. Fatigue fractures should be considered in conscripts seeking medical advice for complaints of musculoskeletal pain, and taken into consideration in planning military and other physical training programs.

## Background

Intense or recently intensified physical activity can lead to stress fractures, overuse injuries that are commonly encountered in sports and military medicine. Stress fractures commonly occur in long-distance runners, dancers, and other athletes, as well as in military trainees and soldiers [1–3]. Stress fractures are generally classified as either fatigue fractures, which occur in normal bone due to loading with abnormal forces, or insufficiency fractures, which occur in abnormal bone after loading with normal forces [1–3].

The risk of fatigue fracture is often related to the amount of physical activity, such as running, marching with loads, and walking [4, 5]. Not all factors that predispose to the development of stress fractures, however, are known. For example, some studies suggest a possible genetic predisposition for stress fractures [6, 7]. A low serum 25(OH)D concentration is also associated with bone fatigue fractures [8].

Bone stress injuries are categorized as either high-risk or low-risk, depending on the potential adverse sequelae and long-term morbidity [9]. A high percentage of patients with a displaced femoral neck fatigue fracture (high-risk) experience long-term morbidity, while patients with a non-displaced femoral neck fatigue fracture (low-risk) are not predisposed to long-term morbidity if dislocation is avoided [10, 11].

Stress fractures often lead to military discharge during basic training [12]. Recruits developing a stress fracture during their basic training period are at higher risk for stress fractures during subsequent training [13]. Such stress fractures may also increase the number of sick days, thereby disrupting military training. In the military, it is very important to understand not only the benefits of physical training, but also the short-term risks. Even relatively minor injuries might be costly in terms of lost training time and reduced combat readiness of soldiers [14]. Good physical fitness resulting from physical training is considered an essential element of readiness, but may lead to an increased incidence of training-related injuries.

Despite numerous studies of conscripts, recruits, and military personnel, population-based studies are required to better elucidate the incidence and risk factors for developing stress fractures. In the present cohort study, we evaluated the incidence, risk factors, and consequences of fatigue fractures, as well as the effectiveness of applied preventive measures among male conscripts during their compulsory military service period in Finland.

## Methods

All Finnish males are required to serve in the military, with approximately 80% of each generation performing their service annually. Each year in January or July, new groups of conscripts begin their training. The majority of conscripts fulfil their service requirements at 19 to 20 years of age, although the age ranges from 18 to 29. The military training requirement is 180 days for conscripts with rank and file duties, 270 days for those with special training, and 362 days for officers and highly trained conscripts.

Subjects of the present study were 5000 conscripts randomly selected from the Finnish population registry, 1000 each born in 1969, 1974, 1979, 1984, or 1989, that were compared with each other over time. Military service documents were confirmed for 4327 of the men; of these, 298 were excluded because of a change in the service selection after entering the military or discharge for health reasons. Data for 4029 men were thus included in the analysis. The study received approval from the Institutional Review Board of the Finnish Defence Forces and the Ethics Committee of Helsinki University Hospital (267/13/03/09).

Basic training lasts 8 weeks and is intended to prepare the conscripts both mentally and physically for the military. Instruction is provided regarding the basic knowledge, performance, and skills required for military service. Physical training accounts for an average of ~ 17 h per week and includes combat skills, marching, and other physically demanding training such as close-order drills. Sport-related physical training (SRPT) accounts for ~ 6.5 h per week. Physical training proceeds progressively mainly through SRPT to help conscripts reach their maximal performance capacity by the end of the military service period. To reduce the risk of stress injuries while ramping up the physical training load, training is lightest during the first 2–6 weeks of training and individualized as much as possible [15–17]. SRPT is increased to support physical activity loading in the middle 8 weeks of service when there are fewer physically demanding training activities. In the troop training phase (latter 8 weeks), additional physical loading is provided by combat exercises and combat shooting camps, which often last several days at a time, and exercise training is intended mainly to promote recovery and mental relaxation.

Military training requires carrying heavy loads, such as combat dress and gear weighing ~ 25 kg, personal weapons, and other required equipment. The SRPT and physical training demands during the earlier phases help conscripts to cope with the greater demands of battlefield training and to increase their resistance to stress injury. Details of the basic training are available elsewhere [18].

Prior to entering military service, the Finnish Defence Forces require all conscripts to complete a pre-information questionnaire regarding socio-economic factors, health self-assessment, baseline health behaviour, school success, education level, father’s occupation, urbanization ranking of place of residence, physical activity level, prior sporting activities, sports club membership, participation in competitive sports, significant health factors, previous sports injuries, orthopaedic surgery, regular medication, chronic disease, impairment or disability, and presence of pain in seven different anatomic regions in the last month. Additionally, the questionnaire includes questions about the use of tobacco and alcohol, and frequency of drunkenness. Conscripts are asked how they think they compare with similar age mates and what they think about the physical demands placed on a soldier. The details of the questionnaire were reported previously [19–23]. All conscripts who participated in the present study served as either Class A (full combat or field troop training) or Class B (lightened or service training) [24].

Any visit to the garrison health care centre by the conscripts to seek medical advice was coded according to International Classification of Diseases (ICD)-9 or ICD-10 diagnosis codes indicating stress fracture. Only fatigue fractures were included in the present analysis. For each visit to the garrison health care centre, the medical reports were reviewed and the following data were recorded: examination date, symptoms, diagnosis, and resulting exemptions from duty. In all but one case, a plain radiograph was obtained to confirm the diagnosis. In our institution, when the diagnosis of stress fracture is uncertain on the basis of a plain radiograph, scintigraphy or magnetic resonance imaging (MRI) is performed. The imaging modalities and diagnostic criteria remained the same for the entire study.

### Statistical analysis

The results are provided in the tables as means with standard deviations, and reported in the text as absolute and relative (%) counts. The t-test and chi-square test were applied to analyse the significance of differences between groups. Statistical analysis of the time-to-event was performed using the product-limit approach by applying the bootstrap 95% confidence interval [CI] of the cumulative incidence function. A multivariable forward stepwise Poisson regression model was used to determine the independent effect of fatigue fractures. The Poisson regression model was tested using the goodness-of-fit test and assumptions of overdispersion in the Poisson model were tested using the Lagrange multiplier test. Analyses were performed using the Stata 14.1, StataCorp LP (College Station, TX, USA) statistical package.

## Results

**Fig. 1:**
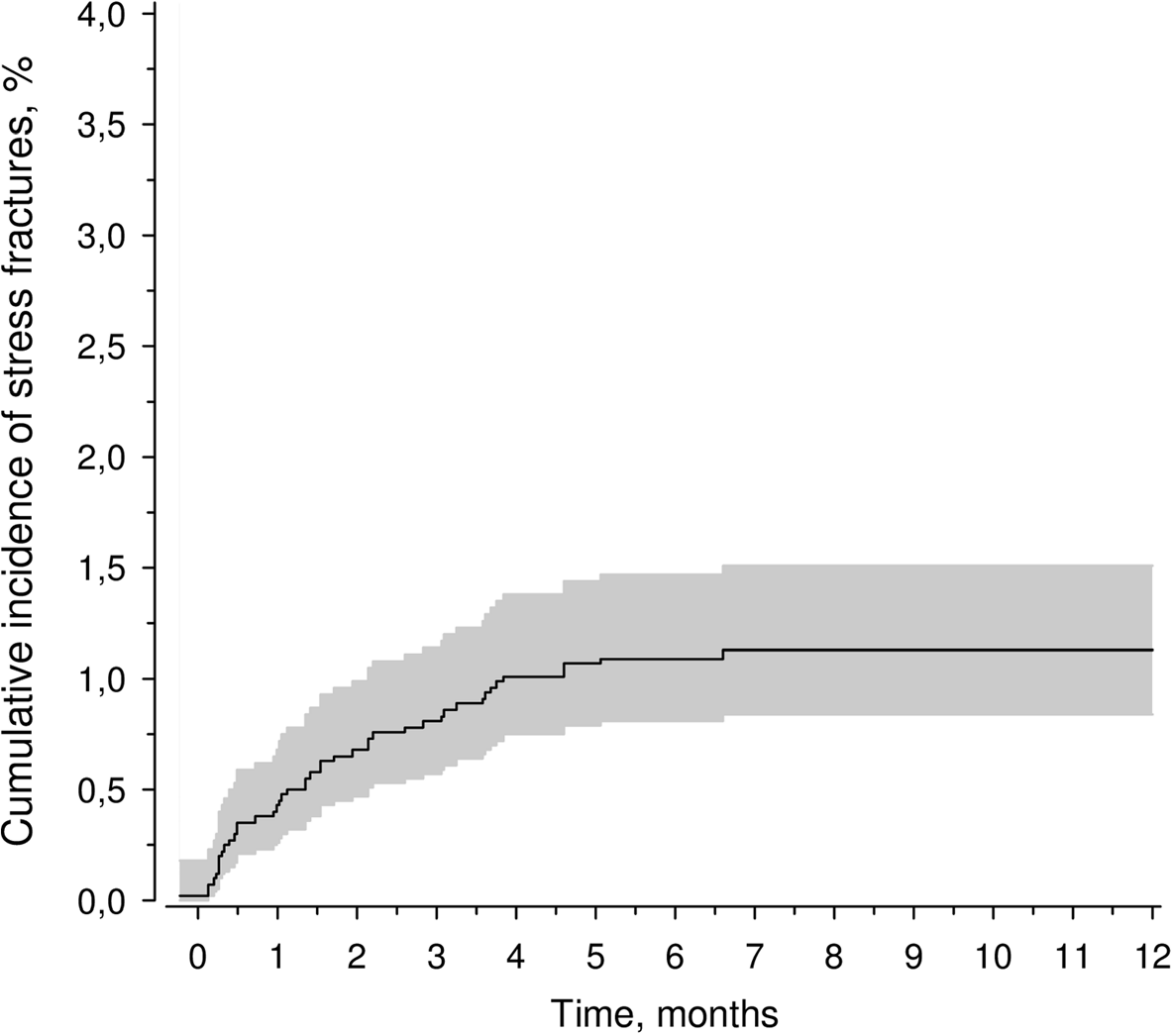
Cumulative incidence of stress fractures during military service

Fatigue fractures were detected in 44 (1.1%) of the study population (*n* = 4029 men; Tables 1 and 2). The incidence of fatigue fractures during military service was 1.27 (95% CI: 0.92–1.70) per 1000 follow-up months (Fig. 1). According to the medical reports, one of the stress fractures was diagnosed 1 week prior to beginning military service. None of conscripts in the study developed insufficiency fractures. Diagnosis was based on clinical examination (patient history and palpable tender bony mass) alone in 1 case; clinical and radiological investigation in 38 cases; clinical findings, plain radiographs, and MRI in 2 cases; and clinical findings, plain radiographs, and scintigraphic examination in 3 cases. Thus, in addition to radiographs, MRI or scintigraphy was needed to confirm the diagnosis in five cases.

**Table 1:** Demographic and clinical characteristics recorded from the questionnaire regarding the presence of fatigue fractures

	Stress fracture	No stress fracture	*P* value [statistics]
N	44	3985	
Age (y), mean (SD)	19.2 (0.6)	19.2 (1.1)	0.82 [t = 0.22]
Class A of service, n (%)	43 (98)	3683 (92)	0.18 [x^2^ = 0.18]
Body mass index (BMI), mean (SD)	22.7 (3.9)	23.3 (3.8)	0.32 [t = 1.00]
Smoking, n (%)	9 (24)	1152 (34)	0.23 [x^2^ = 1.45]
Comprehensive school only, n (%)	27 (63)	2318 (58)	0.56 [x^2^ = 0.33]
Self-reported high physical activity (more than twice a week, causing sweating and breathlessness) before military service, n (%)	9 (20)	1299 (38)	0.017 [x^2^ = 5.71]
Diseases and injuries before entering service, n (%)			
F00-F99 Mental and behavioural disorders	1 (2)	177 (4)	0.49 [x^2^ = 0.48]
H00–H59 Diseases of the eyes and adnexa	11 (25)	1091 (27)	0.72 [x^2^ = 0.12]
J00–J99 Diseases of the respiratory system	3 (7)	589 (15)	0.14 [x^2^ = 2.20]
L00–L99 Diseases of the skin and subcutaneous tissue	1 (2)	229 (6)	0.32 [x^2^ = 0.98]
M00–M99 Diseases of the musculoskeletal system	5 (11)	343 (9)	0.52 [x^2^ = 0.41]
Self-reported symptoms on admission, n (%)			
Injuries	4 (9)	414 (10)	0.78 [x^2^ = 0.08]
Symptoms of the musculoskeletal system	1 (2)	353 (9)	0.12 [x^2^ = 2.36]
Respiratory symptoms	5 (11)	836 (21)	0.12 [x^2^ = 2.44]
Symptoms of the gastrointestinal tract	0 (0)	88 (2)	0.32 [x^2^ = 0.99]
Mental symptoms	3 (7)	138 (3)	0.20 [x^2^ = 1.46]
Headache	5 (11)	211 (5)	0.076 [x^2^ = 3.16]

**Table 2:** Number of fatigue fractures (*n* = 44) based on anatomical location

Location	n
Metatarsal bones	17
Tibia	16
Calcaneus	2
Femoral shaft	2
Upper part of femur	2
Femoral neck	1
Upper part of tibia	1
Both femurs, upper part	1
Femur and calcaneus	1
Tibia and metatarsal bone	1

**Fig. 2:**
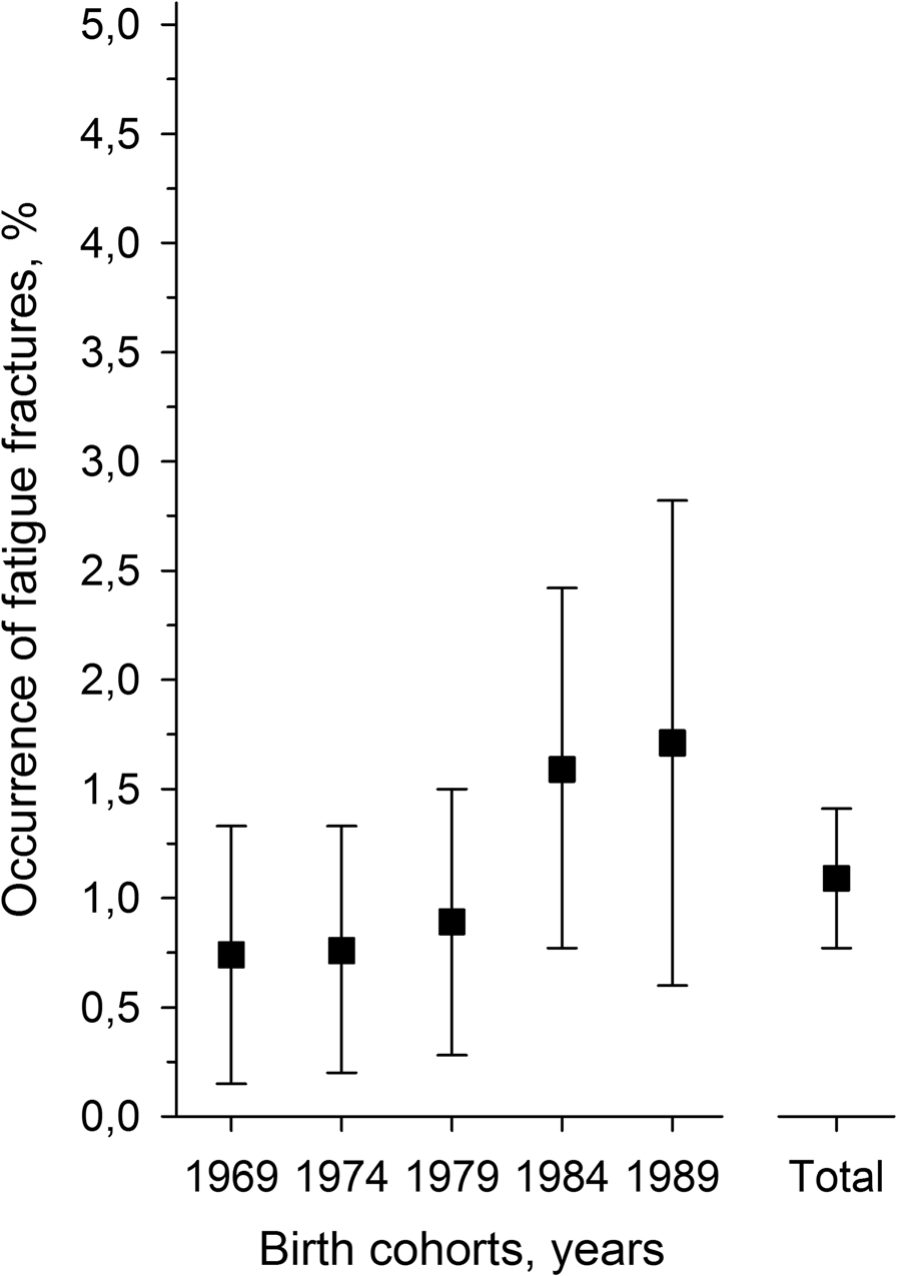
Occurrence of fatigue fractures. Birth cohorts did not differ significantly (*p* = 0.21)

**Fig. 3:**
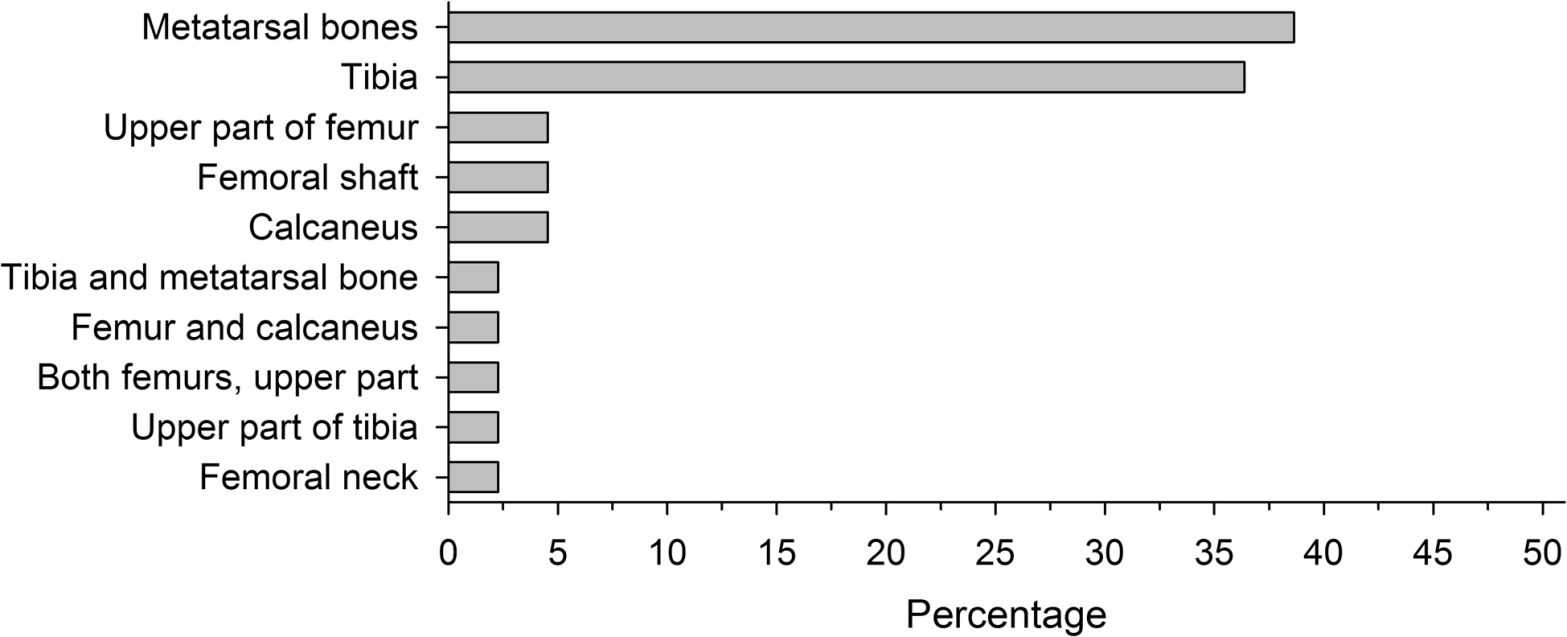
Anatomical location of stress fractures in lower extremities

The incidence was similar among the different age cohorts, although the prevalence tended to increase slightly in the two most recent cohorts (Fig. 2). The age cohorts did not differ significantly in the incidence of fatigue fractures (*p* = 0.21). All 44 fatigue fractures were located in the lower extremities (Fig. 3). The majority of fatigue fractures, 33 (75%) were located in the tibial shaft or metatarsals. In three patients, two simultaneous stress fractures were detected: one had both tibial and metatarsal fatigue fractures, another had bilateral fatigue fractures in the upper part of the femoral shaft, and a third had a femoral shaft fracture and a fatigue fracture of the calcaneal bone (Table 2). Most fatigue fractures occurred within the first 4 months of military service (Fig. 1).

Conscripts with fatigue fractures lost a total of 1359 (range 10–77) active military training days due to exemptions from duty. Diagnosis of fatigue fracture led to reclassification by the garrison physician of two conscripts serving in Class A (full combat or field troop training) to Class B conscripts (lightened or service training). In addition, three conscripts were classified as Class E conscripts; i.e., military service had to be discontinued until a physician deemed them capable of completing their remaining military service.

Conscripts who reported regular (> 2 times/week) physical activity (i.e., physical exercise causing sweating and breathlessness) before entering military service had significantly fewer bone fatigue fractures (*p* = 0.017, Table 1). Other measured parameters, such as body mass index, did not significantly affect the incidence of stress fractures. All of the variables shown in Table 1 were included in the multivariate forward Poisson regression model (Table 3), and only physical activity was a significant explanatory variable [IRR = 0.41 (95% CI: 0.20 to 0.85)].

**Table 3:** Poisson regression models for the risk of fatigue fractures in young Finnish men performing their compulsory military service

	Univariate IRR (95% CI)	Multivariate^a^IRR (95% CI)
Age (y) of military service	0.95 (0.72 to 1.26)	
Class A of service	3.30 (0.45 to 23.94)	
BMI	0.96 (0.88 to 1.05)	
Smoking	0.68 (0.32 to 1.44)	
Comprehensive school only	0.75 (0.41 to 1.40)	
Self-reported pre-military service high physical activity	0.41 (0.20 to 0.85)	0.41 (0.20 to 0.85)
Diseases and injuries		
F00-F99 Mental and behavioural disorders	0.45 (0.06 to 3.27)	
H00–H59 Diseases of the eyes and adnexa	0.89 (0.45 to 1.76)	
J00–J99 Diseases of the respiratory system	0.43 (0.13 to 1.40)	
L00–L99 Diseases of the skin and subcutaneous tissue	0.40 (0.05 to 2.88)	
M00–M99 Diseases of the musculoskeletal system	1.46 (0.57 to 3.70)	
Self-reported subjective symptoms on admission		
Injuries	0.90 (0.32 to 2.52)	
Symptoms of the musculoskeletal system	0.25 (0.03 to 1.82)	
Respiratory symptoms	0.48 (0.19 to 1.23)	
Mental symptoms	2.09 (0.65 to 6.74)	
Headache	2.38 (0.93 to 6.04)	

^a^Forward stepwise selection. Only those variables shown which entered the model

## Discussion

The findings of the present study suggest that engaging in highly physical activity, i.e., physical exercise causing sweating and breathlessness, more than twice a week before entering military service, protects young adult men from fatigue fractures during military service. To the best of our knowledge, this is the first report that regular physical activity prior to entering military service diminishes the incidence of fatigue fractures in young adult men during military service. Thus, engaging in regular, continuous, highly physical activity, before entering military service appears to be an important preventive measure that should be emphasised.

The low incidence of fatigue bone fractures, 1.1%, in the present cohort study indicates that the physical training load of the study population was not too heavy for the bones of a large majority of the conscripts. According to recent reports, the incidence of stress fractures in military recruits or conscripts ranges between 1.6 and 5.0% [25–27]. The low incidence of stress fractures in the present study indicates that the physical training level during compulsory military service was not too strenuous. Stress fractures should be considered, however, when conscripts present to the clinic with complaints of musculoskeletal pain, and taken into account when planning military and other physical training programs.

In the present study, conscripts suffering from fatigue fractures lost a total of 1359 (range 10–77) active military training days due to exemptions from duty. Stress fractures also caused service class changes. Stress fractures in athletes and military trainees results in a loss of active training days, and may be associated with adverse consequences and long-term morbidity [8, 28]. Physicians treating military trainees should keep these conditions in mind when military trainees complain of musculoskeletal pain. Some earlier epidemiologic studies suggested that the incidence of stress fractures can be reduced by altering modifiable risk factors of stress fractures, such as training volume, running distance, and possibly the use of shock-absorbent insoles in military boots [29, 30].

Our study has a number of strengths. First, we analysed five large age cohorts resulting in a total 4029 conscripts. The 5-year interval between the cohorts allowed us to evaluate the incidence over time. Furthermore, the medical examination and treatment in military health care centres and military hospitals was consistent for every conscript according to the policy of the Finnish Defence Forces. The present study also has some limitations. The present study is a registry-based study, and thus does not allow for exploration of theoretical or biological explanations for the findings. Only MRI can reveal the early stages of a stress fracture, i.e., bone pathology. According to the standard policy of the Finnish Defence Forces, MRI was not used to identify low-risk stress injuries, such as those in the metatarsal bones, because they can usually be detected on plain radiography. The use of MRI to evaluate every clinically suspicious bone stress injury would likely increase the accuracy of the diagnosis of stress fractures and reveal more bone stress injuries, but this would not reflect normal clinical practice for examining and treating fatigue fractures.

The majority (75%) of fatigue fractures detected in the present cohort study were located in the tibial shaft or metatarsals. This finding is consistent with those of previous studies of both Finnish athletes and conscripts [31, 32]. In a previous study of US Marine Corps recruits, bone stress injuries were less commonly detected in the pelvis, hip, thigh, and knee [33]. In running athletes, metatarsal shaft stress fractures are the most common type, representing up to 20% of stress fractures in the athletic population [34]. These fractures reportedly account for 38% of all stress fractures of the lower limb [12]. Among soldiers, the anatomical distribution of stress fractures differs for Israeli elite troops, with a higher incidence of tibial and femoral stress fractures during the first and second phases of training, and a higher incidence of metatarsal stress fractures in the third phase [35].

## Conclusions

The main finding of the present study is that highly physical activity, physical exercise causing sweating and breathlessness more than twice a week before entering military service may protect young adult men from fatigue fractures. Thus, regular and recurrent high-intensity physical activity before entering military service seems to be an important preventive measure against developing fatigue fractures. Engaging in regular continuous highly physical activity before entering military service should be encouraged and emphasised.

## Abbreviations

ICD: International Classification of Diseases
SRPT: Sport-related physical training
